# Synthesis and stabilization of black phosphorus and phosphorene: recent progress and perspectives

**DOI:** 10.1016/j.isci.2021.103116

**Published:** 2021-09-13

**Authors:** Yonghong Zeng, Zhinan Guo

**Affiliations:** 1 Institute of Microscale Optoelectronics, International Collaborative Laboratory of 2D Materials for Optoelectronics Science and Technology, College of Physics and Optoelectronic Engineering, Shenzhen University, Shenzhen 518060, P. R. China

**Keywords:** Physics, Nanotechnology, Materials synthesis

## Abstract

Two-dimensional black phosphorus (BP) has triggered tremendous research interest owing to its unique crystal structure, high carrier mobility, and tunable direct bandgap. Preparation of few-layer BP with high quality and stability is very important for its related research and applications in biomedicine, electronics, and optoelectronics. In this review, the synthesis methods of BP, including the preparation of bulk BP crystal which is an important raw material for preparing few-layer BP, the popular top-down methods, and some direct growth strategies of few-layer BP are comprehensively overviewed. Then chemical ways to enhance the stability of few-layer BP are concretely introduced. Finally, we propose a selection rule of preparation methods of few-layer BP according to the requirement of specific BP properties for different applications. We hope this review would bring some insight for future researches on BP and contributes to the acceleration of BP's commercial progress.

## Graphical abstract

**Figure:**
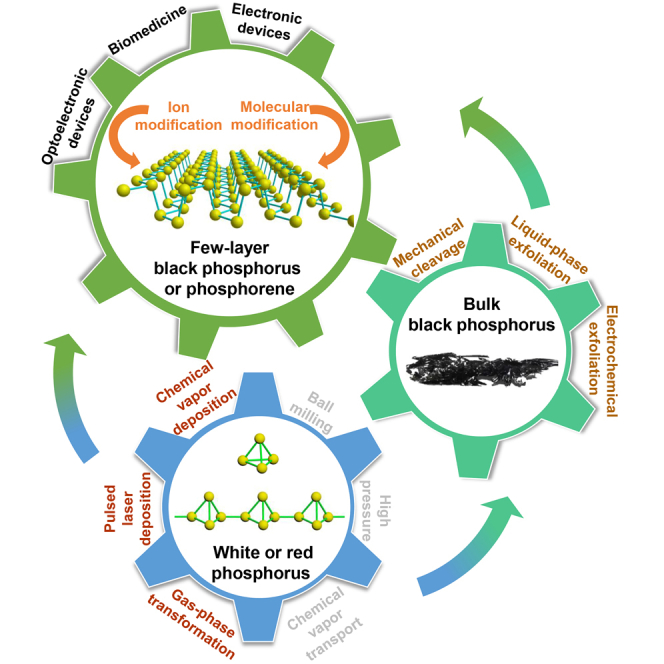

## Introduction

In 2014, the research teams of Ye (Liu et al., 2014) and Zhang (Li et al., 2014) independently reported that black phosphorus (BP), as a new elemental two-dimensional (2D) material, exhibits excellent electrical properties (mobility of ∼1000 cm^2^ V^−1^ s^−1^ and on/off current ratio of up to 10^5^ at room temperature) and anisotropic characteristics with a tunable bandgap (0.31 eV–1.0 eV), imparting BP with great potential for application in electronic and optoelectronic devices. The bandgap of 2D BP sits just between that of graphene (0 eV) (Novoselov et al., 2004) and transition-metal dichalcogenides (TMDs) (1.0 eV–2.0 eV) (Butler et al., 2013; Radisavljevic et al., 2011; Wang et al., 2012). Its photoresponse in the infrared (IR) range, together with its highly anisotropic light-mater interaction, makes BP an exciting new research topic in optoelectronics, photonics, and biomedicine based on 2D materials. In the past 5 years, more than 1000 articles related to BP have been published per year, indicating the high level of enthusiasm devoted to BP-related research.

The reasons for the widespread research interest in BP can be summarized as follows: i) The super high drug-loading capacity (up to 950% in weight) and excellent photothermal conversion efficiency (28.4%) benefitting from BP's puckered honeycomb structure makes BP appealing for applications in controllable drug delivery and cancer therapy (Chen et al., 2017b; Fan et al., 2018; Sun et al., 2015); ii) BP is a good candidate for developing high-speed flexible electronic devices because of its high carrier mobility and appropriate on-off current ratio (Liu et al., 2015; Xia et al., 2014a; Xu et al., 2019); iii) The direct bandgap and saturable absorption nature of BP would facilitate its application in ultrafast laser photonics such as mode locking lasers (Chen et al., 2015b; Lu et al., 2015; Zhang et al., 2019b), modulators (Guo et al., 2019a; Sun et al., 2016b), and photodetectors (Xia et al., 2014b). Although BP has so many excellent properties and application prospects, two main disadvantages hinder its practical application. One is the poor stability of 2D BP in ambient conditions. Despite bulk BP being the most stable of the phosphorus allotropes, the oxygen and moisture in the air readily reacts with BP and destroys its crystal structure when it is exfoliated into a few-layer structure (Favron et al., 2015; Island et al., 2015; Zhou et al., 2016). The other is the lack of synthesis methods for large-area high-quality few-layer BP (Castellanos-Gomez, 2015; Khurram et al., 2020). Currently, few-layer BP is mainly produced by a top-down processing of bulk BP crystals, which is hard to apply as a common method of mass production of few-layer BP with ideal interfaces.

This review summarizes the majority of reported synthesis methods for BP crystal, few-layer BP, and BP quantum dots, as well as the strategies to enhance the stability of BP. It is our intention to bring insight for future BP research and contribute to the acceleration of BP's commercial progress.

## Fundamental basic structure and properties of BP

### Fundamental basic structure of BP

**Figure 1:**
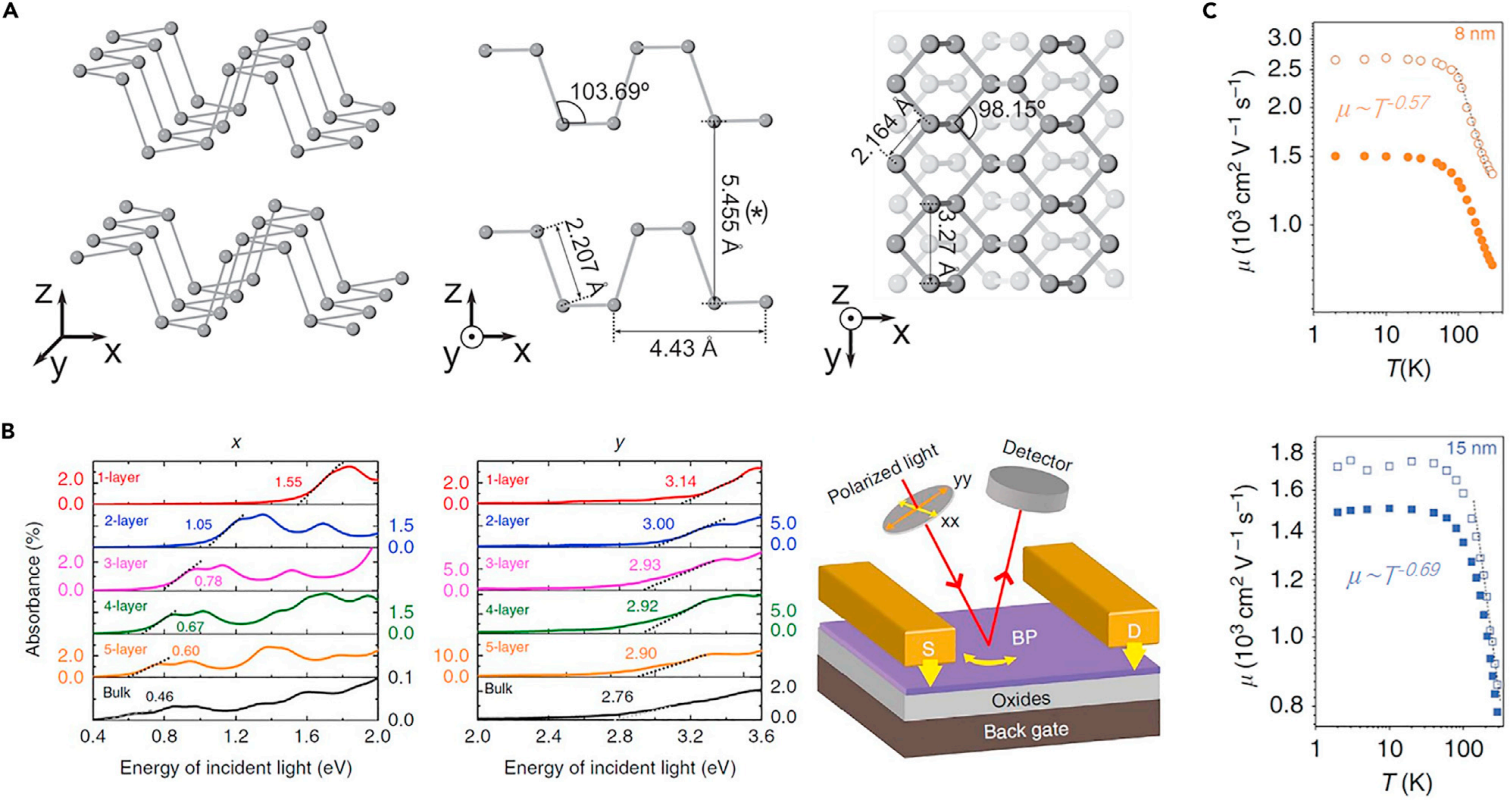
Crystalline structure and properties of black phosphorus (A) Schematic diagram of the crystalline structure of BP from a three-dimensional (left), side (middle), and top (right) view. (B) Optical absorption spectra of BP with different layers under polarized light in the *x*-direction (left) and *y*-direction (middle). Scheme for optical absorption spectroscopy to determine the orientation of few-layer BP (right). (C) μ_FE_ (open circles) and μ_H_ (solid dots) as a function of temperature at a gate voltage of 70 V for BP samples of 8 nm (top) and 15 nm (bottom).

Phosphorus, as one of the most abundant elements on earth, usually has various allotropes, such as red phosphorus, white phosphorus, purple phosphorus, and black phosphorus. Compared with other phosphorus allotropes, BP exhibits higher structural stability owing to its unique orthorhombic crystal structure. Much similar to graphite, bulk BP is composed of many stacked planes of monolayer BP (otherwise known as phosphorene) bonded by weak van der Waals (vdWs) forces, whose interlayer spacing is ∼0.55 nm. Each BP monolayer is composed of sp^3^ hybridized phosphorus atoms which results in a nonplanar folded hexagonal structure, forming a puckered honeycomb structure. Unlike the P_4_ tetrahedral structure of white phosphorous, the hybridization of phosphorene breaks the single P_4_ bond and forms two different bond angles of 98.15° and 103.69°, which are closer to the perfect tetragonal structure with an angle of 109.50°, making BP more stable (Carvalho et al., 2016). As shown in Figure 1A, phosphorene has two crystal directions in the plane, called the armchair direction (or *x* axis) and the zigzag direction (or *y* axis), which are orthogonal and parallel to the corrugation direction, respectively. The lattice parameters of BP are 4.43 Å and 3.27 Å along the armchair direction and the zigzag direction, respectively. The two different bond lengths of BP are ∼2.16 Å in-plane (connecting the phosphorus atoms within the same layer) and ∼2.21 Å out-of-plane (connecting the phosphorus atoms between adjacent layers) (Castellanos-Gomez et al., 2014). BP has the following unique electrical and optoelectrical properties closely relating to its unique basic structure.

### Properties of BP

As a semiconductor material, BP exhibits direct bandgap characteristics regardless of the number of layers (Churchill and Jarillo-Herrero, 2014) and a layer-dependent bandgap, whose value is in the range of 0.10–0.35 eV for bulk BP and 1.0–1.7 eV for monolayer BP, as demonstrated by theoretical calculations and experimental results (Li et al., 2017a; Liu et al., 2014; Rudenko and Katsnelson, 2014). Although there are differences in the reported values of the BP bandgap, a similar layer-dependent trend is observed: the bandgap decreases monotonically with the number of layers increasing, which is attributed to the vdWs interaction resulting in the band splitting. In addition to the number of layers, the bandgap of BP can also be altered by strain engineering (Fei and Yang, 2014), chemical functionalization (Li et al., 2016), external electric field (Zhang et al., 2017a), and doping (Jing et al., 2015).

BP exhibits a wide range of light absorption from ultraviolet (UV) to IR (Buscema et al., 2014; Guo et al., 2016; Tran et al., 2014) owing to the adjustable bandgap of BP itself while also showing anisotropic light absorption properties due to its highly anisotropic puckered honeycomb structure, as demonstrated by its strong dichroism (Wang et al., 2015; Xia et al., 2014a). Specifically, the absorption coefficient of BP depends on the polarization state of incident light, with BP absorbing incident light of different degrees of polarization at different rates in different directions (Qiao et al., 2014), as illustrated in Figure 1B.

As a further excellent feature that has attracted the attention of many researchers to this new material, BP exhibits a high carrier mobility (∼1000 cm^2^ V^−1^ s^−1^ at room temperature) compared with TMDs (Long et al., 2019). From the formula $\mu =q\tau /m^{*}$, where *q* is the basic electron charge constant, it can be seen that the carrier mobility *μ* is related to the scattering time τ and the effective mass $m^{*}$ (Yi et al., 2017). BP exhibits anisotropic mobility, which is mainly ascribed to the anisotropy of $m^{*}$, as ultrafast IR spectroscopy analysis of BP flakes reveals that τ is almost isotropic with scattering direction (Ge et al., 2015). In 2014, Qiao et al. disclosed that the effective masses of holes and electrons for phosphorene in the zigzag direction were 6.35$m_{0}$ and 1.12$m_{0}$ respectively, in which $m_{0}$ is the free electron mass. On the contrary, in the armchair direction, the effective mass of holes was 0.15$m_{0}$ and the effective mass of electrons was 0.17$m_{0}$ (Qiao et al., 2014). It can be seen from the aforementioned formula that a carrier with a large $m^{*}$ has a low *μ* meaning that it is not conducive to carrier transmission. Temperature also affects the mobility of BP. As shown in Figure 1C, the field-effect mobility (μ_FE_) and Hall mobility (μ_H_) of high-quality BP field-effect transistors (FETs) increases with decreasing temperature and reaches saturation at low temperatures (*T* < 80 K). At higher temperatures (*T* > 80 K), the mobility obeys the inverse power law of $\mu ∼T^{-\gamma }$, in which γ is measured to be 0.57 and 0.69 for 8- and 15-nm-thick BP, respectively. The mechanism can be explained by electron-phonon scattering for higher temperatures (*T* > 80 K) and impurity scattering for low temperatures (*T* < 80 K) (Chen et al., 2015a).

## Synthesis of bulk BP crystals

High-quality bulk BP is an important source for preparing few-layer BP; hence, it is important to understand the current synthesis methods of bulk BP. In the following, we will introduce several methods of synthesizing bulk BP crystals: high-pressure preparation, mercury catalysis, liquid bismuth recrystallization, and chemical vapor transport (CVT).

**Figure 2:**
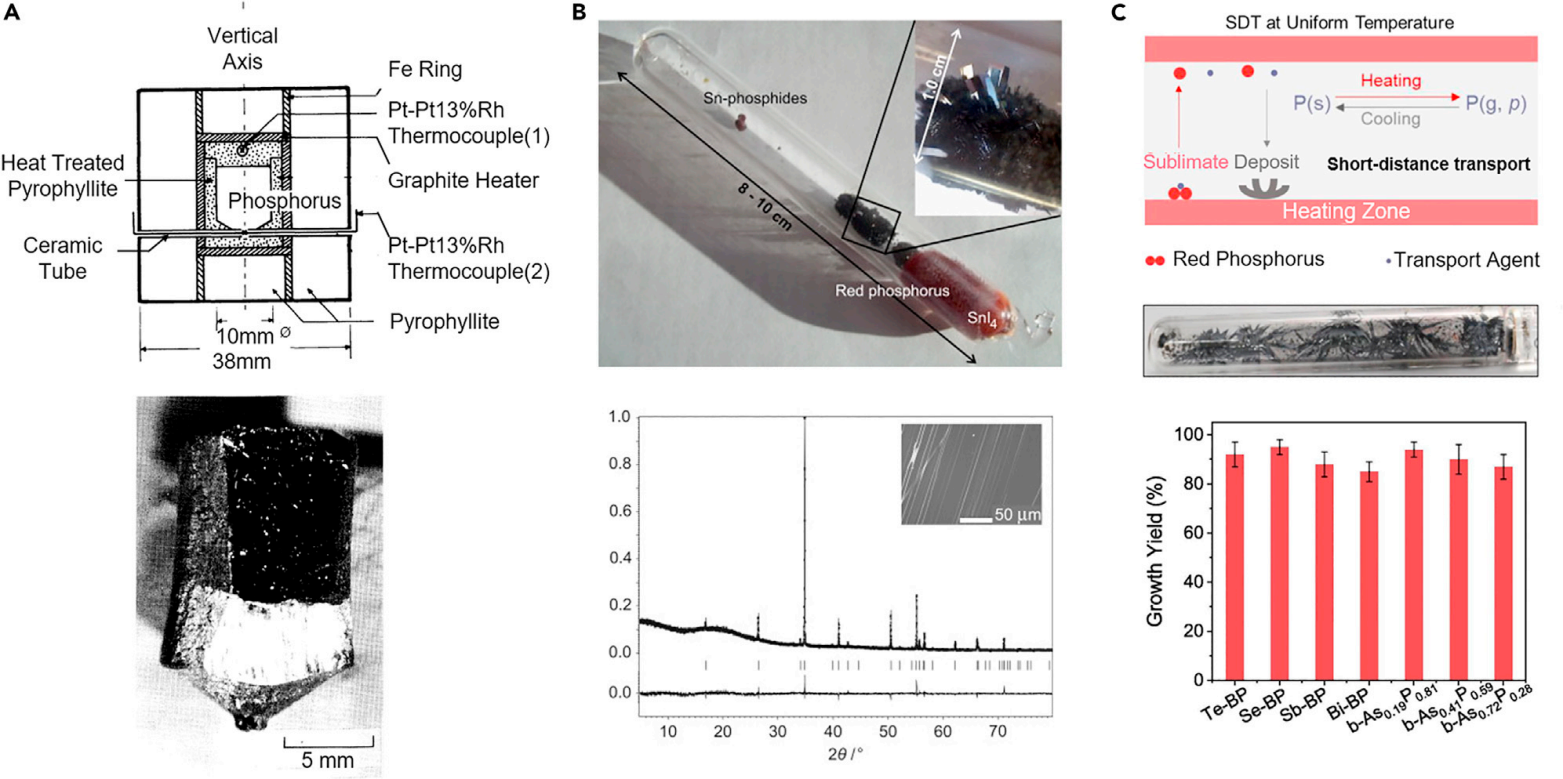
Synthesis of bulk black phosphorus crystals (A) (Top) A cross section of cubic cell designed for growth of bulk BP under high pressure and high temperatures. (Bottom) An ingot of synthesized BP crystal. (B) (Top) A silica ampule containing CVT-grown bulk BP. (Bottom) XRD from CVT-grown bulk BP revealing no crystalline impurities and the scanning electron microscopy (SEM) image of the prepared BP (inset). (C) Schemes of SDT at uniform temperature (top). Photograph of the as-grown product in the SDT growth (middle). Growth yields of doping BP (bottom).

In 1914, the American physicist Bridgman, who won the Nobel Prize in Physics in 1946 in recognition of his achievements in the field of high-pressure physics, successfully synthesized bulk BP for the first time by heating white phosphorus to a high temperature of 200°C while under a pressure of 1.2 GPa (Bridgman, 1914). Subsequently, the Bridgman method was used to obtain BP crystals for the study of their basic electrical (Akahama et al., 1983), optical (Shibata et al., 1987; Sugai and Shirotani, 1985), and phonon (Ikezawa et al., 1983; Suzuki and Aoki, 1987) properties. High-pressure synthesized BP single crystals with a maximum size of 4 mm in diameter and 5 mm in length were first reported by Shirotani et al. (Shirotani et al., 1981) following the treatment of red phosphorus at 270°C and 3.8 GPa in a reaction vessel composed by pyrophyllite and equipped with a graphite heater and Pt-Pt13%Rh thermocouples, as shown in Figure 2A. Ball milling has also been used to convert red phosphorus into BP by using steel balls in a hardened-steel milling vessel, in which the high temperatures above 200°C and high pressures of about 6 GPa were locally generated (Park and Sohn, 2007).

As alternatives to the Bridgman method and ball milling, which utilize high-temperature and high-pressure conditions, there are also methods that can prepare BP crystal under low-pressure conditions, namely mercury catalysis and liquid bismuth recrystallization. In 1955, as the first method to prepare BP under low pressure (35–45 MPa), white phosphorus was reacted with a mercury catalyst in an autoclave at high temperatures (370–410°C) for several days to finally generate BP crystals (Krebs et al., 1955). Liquid bismuth recrystallization was also used to synthesize needle- or rod-like BP from a phosphorus-bismuth melt (Baba et al., 1989; Brown and Rundqvist, 1965; Maruyama et al., 1981). In the procedure, white phosphorus was dissolved in liquid bismuth and kept at 400°C for 20 h and then slowly cooled to obtain the crystallized BP. However, it should be noted that despite the benefits of low-pressure preparation, these two methods possess the disadvantage that mercury and bismuth are toxic and the prepared BP crystals contain mercury or bismuth to a certain extent.

The final method to be discussed is CVT, which allows synthesis of large-size, high-quality bulk BP single crystals and does not require complicated high-pressure equipment like the Bridgman method or involve toxic materials (Wang et al., 2019a). In 2007, Lange et al first synthesized BP crystals from red phosphorus via CVT with Sn/SnI_4_ as a mineralization additive and small quantities of gold (Lange et al., 2007). In this process, the combined raw materials were heated to a temperature of about 800°C and kept at this temperature for 5–10 days. The reaction was shown to obtain BP single crystal, however, with the presence of additional phases such as Au_3_SnP_7_, AuSn, Sn_4_P_3_, and SnI_4_. Hitherto, the preparation of BP based on CVT has been the focus of much research, on the one hand aiming to reduce the unwanted phases (the so-called impurities) and on the other hand seeking to shorten the reaction time, to obtain high-quality BP single crystals as fast as possible with maximum yield. Köpf et al. have proposed an improved synthesis procedure that costs only about 10 h to synthesize BP crystals of a few millimeters in size from red phosphorus with Sn/SnI_4_ as a mineralization additive (Köpf et al., 2014). Through this improved reaction, the prepared BP single crystal has no other intermediate phases, as verified by the X-ray diffraction (XRD) data shown in Figure 2B. In the absence of other intermediate phases in the prepared BP crystals, Zhao et al. have found that I_2_ and Sn can be used instead of SnI_4_ as mineralization, which will cut down the preparation process of SnI_4_, and reduce the synthesis cost of BP crystals to a certain extent (Zhao et al., 2016b). For another mineralization in the synthesis of BP crystals via CVT, Sn can be completely or partially replaced by some other transition metals, such as Pb (Zhao et al., 2016a). Nevertheless, Sn-I-assisted CVT reactions are still the most common method for synthesizing BP crystals. Recently, an efficient short-distance transport (SDT) method based on CVT to synthesize high-quality BP in which the conversion ratio from red phosphorus to BP can reach up to 98% was reported by Liu et al. Furthermore, the method can achieve doping of BP by Te, Se, Sb, Bi, and As (Liu et al., 2020), as depicted in Figure 2C. Finally, it should be mentioned that CVT is not only the main method for synthesizing bulk BP but also a means for the nucleation and growth mechanism of BP investigation, based on which mechanisms such as vapor-solid-solid mechanism (Li et al., 2017b), phase-induced nucleation and growth mechanism (Zhang et al., 2017b), polymerization-like process (Wang et al., 2019b), and the energy change process based on theoretical calculations (Pielmeier and Nilges, 2021) have been obtained.

## Preparation of few-layer BP

### Mechanical cleavage

Before the discovery of graphene in 2004 (Novoselov et al., 2004), theoretical calculations showed that 2D materials were not allowed to exist freely at a finite temperature owing to thermodynamic fluctuations (Evans et al., 2006; Venables et al., 1984; Zinkeallmang et al., 1992). However, the fact is that graphene was discovered as the first 2D material through tape-assisted mechanical cleavage proved the stable existence of 2D materials in actual three-dimensional space. Just as graphene may be obtained from graphite via tape-assisted mechanical cleavage, 2D BP may be obtained from bulk BP owing to its composition of stacked planes of monolayer BP bonded by weak vdWs forces. The specific process is as follows: BP flakes are adhered to a piece of tape which is repeatedly pressed onto and peeled from a substrate. The few-layer BP samples transferred to the substrate can reach a lateral size of several micrometers and thicknesses in the range of several nanometers to tens of nanometers. In early 2014, the few-layer BP obtained by mechanical cleavage was fabricated into FETs which exhibited a high hole mobility of 286 cm^2^ V^−1^ s^−1^, an on/off ratio of up to 10^4^ at room temperature, and anisotropic transport behavior (Liu et al., 2014). Meanwhile, Li et al. have successfully prepared few-layer BP via mechanical exfoliation to fabricate FETs with high carrier mobility of up to ∼1000 cm^2^ V^−1^ s^−1^ (Li et al., 2014). Although there are disadvantages of low yield and uncontrollable shape, thickness and size of mechanically exfoliated few-layer BP samples which naturally limit applications in biomedicine (Liang et al., 2019; Luo et al., 2019b; Xing et al., 2018), energy storage (Luo et al., 2018; Qiu et al., 2017), and photocatalysis (Khan et al., 2019; Li et al., 2019a), it is currently the most suitable preparation method of large-area, high-quality 2D BP for electronic and optoelectronic devices research.

### Liquid-phase exfoliation

Liquid-phase exfoliation (LPE) refers to solvents intercalating a layered material through sonication or vortex forces, resulting in the expansion or exfoliation of corresponding 2D materials and their suspension in solvent. There are generally three steps for preparation of few-layer BP by LPE: (i) dispersion of the bulk BP in a solvent, (ii) sonication exfoliation, and (iii) centrifugation. However, the crystalline quality of the resulting few-layer BP is not generally high which results in unsatisfactory intrinsic electrical properties. However, the method possesses many advantages, such as solution processability and scalability, and provides a method for the synthesis of hybrid/composite materials, making it an appropriate means to synthesize few-layer BP or its composites for applications in the fields of biology and chemistry.

**Figure 3:**
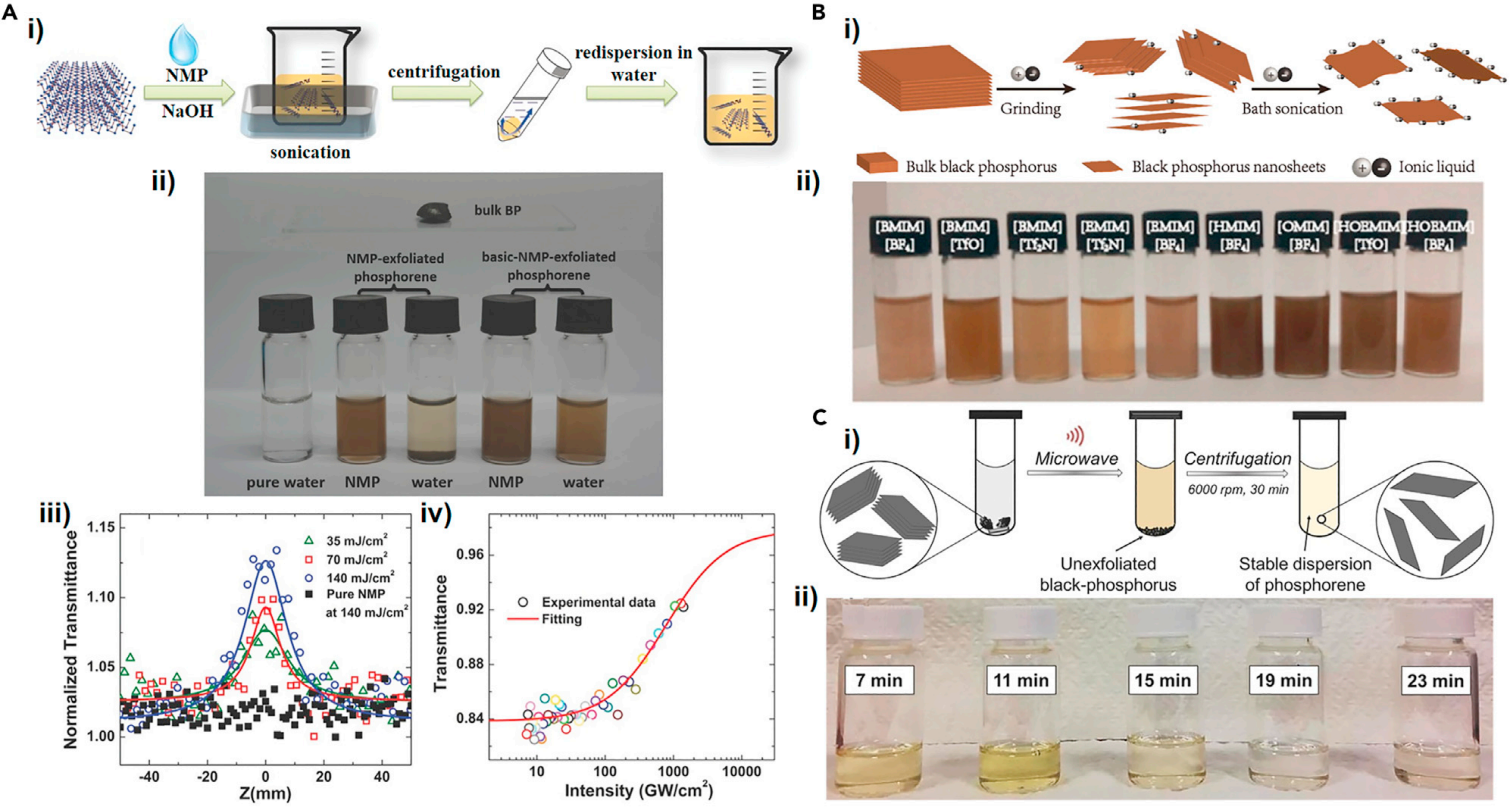
Liquid-phase exfoliation of BP via different strategies (A) (i) Schematic illustration of basic-NMP-exfoliation of bulk BP into phosphorene. (ii) Photographs of bulk BP and phosphorene dispersed in NMP and water. (iii) Z-scan tests of the exfoliated phosphorene at wavelength of 800 nm. (iv) The nonlinear saturable absorption result and its fitting curve of the phosphorene dispersions. (B) (i) Schematic plot of preparation of BP nanosheets via IL exfoliation. (ii) Photograph of few-layer BP solutions using nine different ILs. (C) (i) Schematic for microwave-assisted LPE to obtain few-layer BP solutions. (ii) Photograph of few-layer BP solutions in NMP using different microwave exfoliation times.

In 2014, inspired by the fact that other 2D materials can be prepared by LPE (Coleman et al., 2011; Hernandez et al., 2008; Nicolosi et al., 2013), Brent et al successfully obtained few-layer BP flakes by bath ultrasonication of bulk BP in the N-methyl-2-pyrrolidone (NMP) as an exfoliation medium for the first time (Brent et al., 2014). This method has pioneered the large-scale exfoliation of BP into sheets or even quantum dots, which can be uniformly dispersed in the solvent. However, the disadvantage of this method is that the prepared phosphorene is difficult to stabilize in many conventional solvents such as water, which hinders its application. Based on the effective adsorption of hydroxide radicals on the surface of phosphorene, which improves the stability of BP in water, Guo et al. have demonstrated a basic NMP solvent exfoliation to obtain phosphorene showing a high stability in water, which can be confirmed by zeta potential results (Guo et al., 2015). Figure 3A(i) shows the schematic diagram describing the fabrication process of phosphorene using a basic NMP solvent. As shown in Figure 3A(ii), based on the color of the solutions containing phosphorene prepared from bulk BP with the same weight in each bottle, more efficient exfoliation would be obtained for the basic NMP than for the pure NMP. Besides, the authors provided a method via Raman spectroscopy to fast and effectively achieve *in situ* measurement of layer number of phosphorene (Figure 3A(iii)) and revealed the linear and ultrafast nonlinear optical properties of the basic-NMP-exfoliated phosphorene (Figure 3A (iv)). In 2015, Hanlon et al. exfoliated bulk BP using N-cyclohexyl-2-pyrrolidone (CHP) as a solvent to obtain few-layer BP nanosheets, which exhibited stability for about 200 h in ambient conditions, proving their application potential in gas sensors, saturable absorbers, and reinforcing fillers of composites (Hanlon et al., 2015). The improved stability of CHP-exfoliated BP nanosheets can be explained by the solvation shell on the surface of the nanosheets isolating oxygen and water. In addition to the exfoliation solvents mentioned earlier, the solvents for LPE of BP were extended to other organic solvents, such as dimethylformamide N-cyclohexyl-2-pyrrolidone, isopropyl alcohol, and dimethyl sulfoxide, as summarized in Table 1.

**Table 1:** A comparison of synthetic conditions for few-layer BP with different precursors, solvents, reactions times, lateral sizes, and thicknesses via liquid-phase exfoliation

Precursor	Solvents	Fabrication methods	Time	Product	Lateral dimensions/thickness	Application	Ref.
BP powder	IPA	Ultrasonication	3 h/500 W	QDs	∼5.2 nm/	Hybrid perovskite solar cells	(Chen et al., 2017a)
BP powder	NVP	Ultrasonic, 5°C	5 h/20 W	QDs	∼5 nm/	FETs based on MoS_2_ with BPQDs modification	(Wang et al., 2016)
BP powder	NMP	Probe sonication, bath sonication	3 h/200 W	QDs	2.6 ± 0.9 nm	Saturable absorber device	(Du et al., 2017)
Bulk BP crystals	DMSO	Sonication	15 h/130 W	Nanoflakes	532 nm/15–20 nm	FET	(Yasaei et al., 2015)
Bulk BP crystals	DMSO	Kitchen blender, (1.7–2.1) × 10^4^ rpm	40 min	QDs	2.25 nm/0.58–1.45 nm	photothermal property; humidity sensor	(Zhu et al., 2016)
Bulk BP crystals	IPA	Cup ultrasound sonication, ice bath		Nanosheets	100∼200 nm/2.6 ± 1.5 nm	Drug delivery	(Qiu et al., 2018)
Bulk BP crystals	DMF	Sonication	15 h/130 W	Nanoflakes	190 nm/5.8–11.8 nm	FET	(Yasaei et al., 2015)
BP	Distilled water	Ultrasound irradiation, 20 kHz	30 min/100 W	Nanodots	10 nm/8.7 nm	Cell imaging	(Lee et al., 2016)
Bulk BP crystals	NMP	Ultrasonication	24 h/820 W	Nanosheets	200nm/3.5–5 nm	–	(Brent et al., 2014)
Bulk BP crystals	Basic NMP	Sonication	4 h	Nanosheets	210nm/2.8 ± 1.5nm	Optical saturable absorbers	(Guo et al., 2015)
BP crystals	CHP	Sonication	5 h/750 W	Nanosheets	100nm/2.06 ± 0.18nm	Gas Sensors; saturable absorbers; reinforcing fillers	(Hanlon et al., 2015)
Bulk BP	NMP	bath ultrasonication, 40kHz	4 h/	Nanosheets	∼750 nm/14.4–18.6 nm	all-optical-signal-processing; optical Kerr switcher	(Zheng et al., 2017)
Bulk BP	IPA	Grinding, ice bath sonication	12 h/	Nanosheets		Photodetector	(Chen et al., 2019)
Bulk BP	NMP	Microwave-assisted liquid-phase exfoliation	<12 min	Nanosheets	≤4μm/2-15nm		(Bat-Erdene et al., 2017)

IPA, isopropyl alcohol; NVP, N-vinylpyrrolidone; NMP, N-methyl-2-pyrrolidone; DMSO, dimethyl sulfoxide; DMF, N,N-Dimethylformamide; CHP, N-cyclohexyl-2-pyrrolidone; QDs, quantum dots.

Some research groups have proposed a method to prepare phosphorene using ionic liquids (ILs) as exfoliation medium, which is a category of molten salts at or just above room temperature. ILs have many excellent properties, such as versatile solubility, nonvolatility, high ionic conductivity, nontoxicity, and high thermal stability. The first IL exfoliation trial to synthesize BP nanosheets was proposed by Zhao et al., in 2015, successfully using nine different ILs as exfoliation media and combining mild grinding and weak sonication to obtain monolayer or few-layer BP nanosheets (Zhao et al., 2015), as schematically depicted in Figure 3B(i). The nine corresponding exfoliated BP dispersions are shown in Figure 3B(ii). The thicknesses of the synthesized BP nanosheets were 3.58 nm, 5.50 nm, and 8.90 nm, which verified that few-layer BP nanoflakes could be prepared via LPE using IL solvents. It inevitably takes a few hours by sonication to achieve effective LPE of bulk crystal into 2D sheets, which causes a reduction in the lateral size and increases structural defects. Bat-Erdene et al. have demonstrated a microwave-assisted LPE method to achieve extremely fast production (<12 min) of high-quality few-layer BP flakes (Bat-Erdene et al., 2017). This exfoliation process (Figure 3C(i)) includes two-step microwave exfoliation and centrifugation: Bulk BP immersed in NMP is placed in a microwave system and heated for different time ranging from 4 to 20 min, followed by treatment with another microwave system for 3 min and finally centrifugation. Comparing the color of the solutions from different microwave exfoliation times, as shown in Figure 3C(ii), a more efficient BP exfoliation is observed at 11 min. In further exploration of the rapid exfoliation of BP, the wet-jet milling as a semi-industrial scale approach was applied to achieve ∼100% exfoliation yield within 2 min for preparing few-layer BP. In this process, the bulk BP is ground and dispersed into acetone first, and then the dispersed BP is poured into the wet-jet mill, in which a nozzle with an aperture of 0.3 mm and a piston pass can generate shear forces to exfoliate the dispersed BP. The method can indeed achieve exfoliation of BP in a few minutes, compared with the traditional time-consuming sonication exfoliation (Castillo et al., 2019).

### Electrochemical exfoliation

Electrochemical exfoliation (ECE) refers to current or voltage applied to a working electrode fixed to the bulk-layered crystal material, which drives counterions to the interlayer space, thereby reducing the vdWs force and finally achieving exfoliation of the corresponding 2D material. As a further method to prepare 2D materials in solvent, ECE possesses the advantages of being environmentally friendly, rapid, and convenient (Rabiei Baboukani et al., 2021).

**Figure 4:**
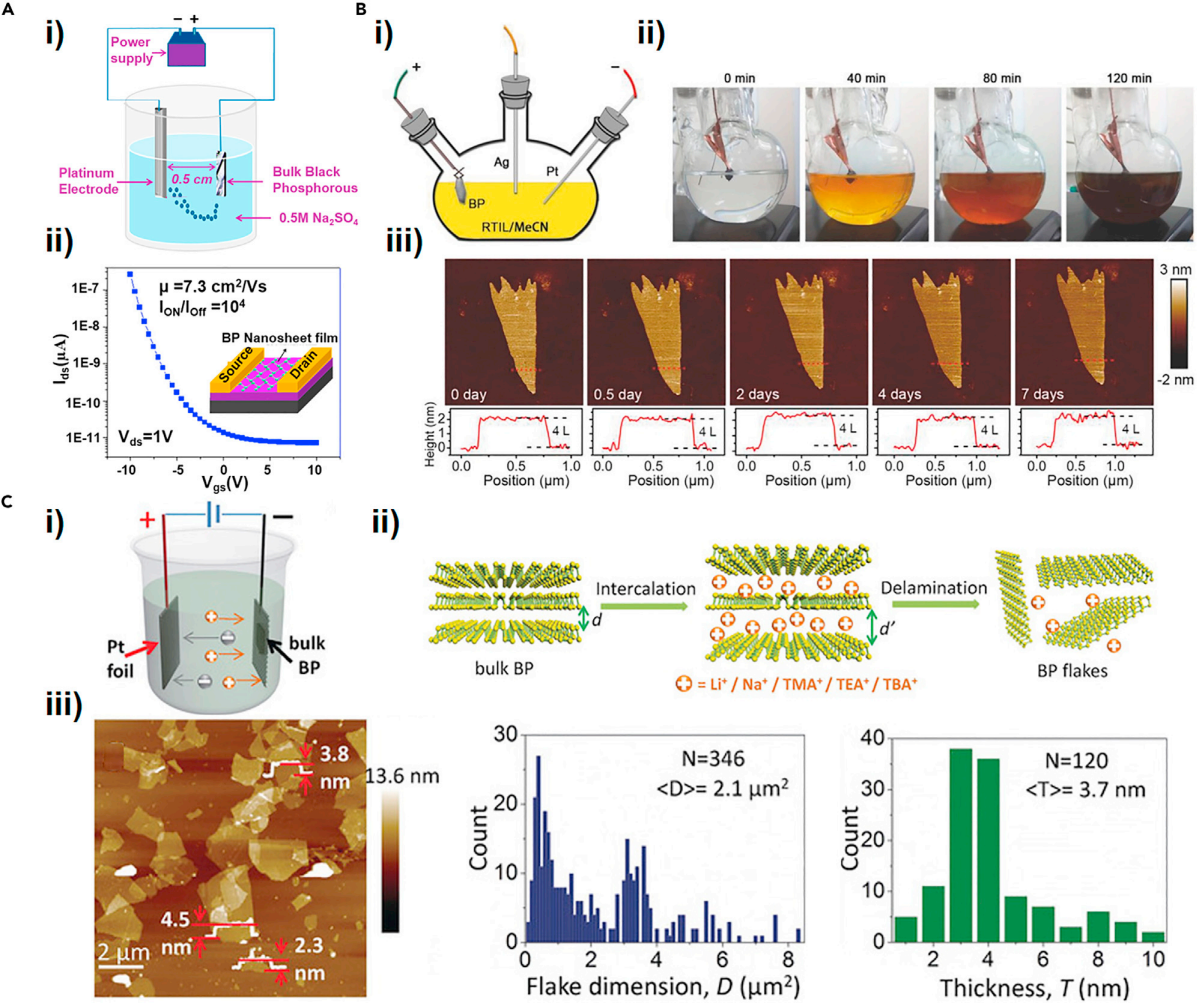
Electrochemical exfoliation of BP via different exfoliation systems (A) (i) Schematic illustration of anodic ECE of BP. (ii) Transfer curves of transistor devices and the schematic diagram of fabricated transistor based on the exfoliated BP, whose substrate is a Si/SiO_2_ (300 nm) wafer (inset). (B) (i) Schematic diagram of experimental device for preparing fluorinated BP, in which RTILs and MeCN are room temperature ionic liquids and acetonitrile, respectively. (ii) Photographs of BP dispersion after different reaction times of the ECE and synchronous fluorination process. (iii) AFM images of a fluorinated BP sample after being exposed to ambient conditions from 0 days to 7 days, and corresponding height profiles measured along the red lines. (C) (i) Schematic illustration of the delamination reaction cell and (ii) procedure. (iii) AFM images of BP flakes obtained by electrochemical delamination (left), and statistical calculation of the dimension (middle) and the height profile (right) of the obtained flakes, in which N is the number of the counted BP.

In 2015, Erande et al. undertook the first trial of ECE to prepare the few-layer BP, by fixing the bulk BP as the anode in an aqueous solution of 0.5 M Na_2_SO_4_ (Erande et al., 2015). The method obtained 2D BP nanosheets with lateral dimensions of 5–10 μm and thicknesses of 1–5 nm, which exhibited excellent field emission characteristics. Figure 4A(i) shows the experimental setup for anodic ECE of BP. In the exfoliation process, oxygen-containing free radicals generated from the electrolysis of water molecules gather at the edge of the bulk BP with the application of voltage. Subsequently, the SO_4_^2−^ ions in the electrolyte quickly enter between the BP layers, thereby weakening their interlayer vdWs interaction. The SO_2_ and O_2_ generated in the electrolyte expand the BP layer, and finally the exfoliated BP nanosheets are obtained (Erande et al., 2016). The exfoliated BP nanosheets possess a yield of 80 wt % and can be applied in FETs which exhibit mobility of ∼7.3 cm^2^ V^−1^ s^−1^ and on/off current ratio of ∼10^4^ as shown in Figure 4A(ii). In 2017, Tang et al. have reported a one-step synthesis strategy for fluorinated BP by IL-assisted anodic ECE and synchronous fluorination. Figure 4B(i) schematically shows the three-electrode electrochemical cell system. After different exfoliation times, the turning of the electrolyte color from transparent to yellow, to orange, and finally to brown showed that BP was effectively exfoliated in this reaction, as depicted in Figure 4B(ii). As shown in Figure 4B(iii), the fluorinated phosphorene exhibited no significant morphological changes after 7 days of exposure to ambient conditions via atomic force microscopy (AFM), which indicated that fluorinated BP has robust ambient stability. Density functional theory calculations have also shown that fluorinated BP can effectively avoid decomposition by O_2_ and will repel oxygen by its electronegativity in comparison with pure BP.

However, the anodic exfoliation will generate oxygen-containing free radicals, unavoidably resulting in the oxidation of the few-layer BP. To address the shortcomings of anodic exfoliation, adopting cathodic exfoliation to prepare large-sized few-layer BP samples in N,N-dimethylformamide (DMF) electrolyte with tetrabutylammonium hexafluorophosphate (TBAP) has been reported by Huang et al. (Huang et al., 2017), and it was found that the BP's layer number can be effectively controlled by the working electrode voltage. The exfoliated BP nanosheets had no surface functional groups and exhibited excellent sodium storage performance. Yang et al. also demonstrated efficient delamination of bulk BP crystal via an ECE method, whose reaction cell and procedure are illustrated in Figure 4C(i) and (ii). Various intercalating agents were systematically studied, such as alkali ions and tetraalkylammonium (alkyl = methyl, ethyl, or n-butyl) cations (Yang et al., 2018). The results of the study showed that the most suitable agent was tetra-n-butylammonium bisulfate, achieving up to 78% exfoliation yield by which flakes with lateral sizes up to 20.6 μm and an average thickness of about 3.7 nm were obtained (Figure 4D(iii)). The exfoliated-BP-based FETs showed an on/off ratio of 1.2 × 10^5^ and hole mobility of 270 cm^2^ V^−1^ s^−1^ at 143 K. In exploring a higher yield of electrochemical exfoliation, Zu et al. reported that high-quality phosphorene with a yield of approximately 93.1% was prepared via cathodic exfoliation (within 5 min) in a propylene carbonate solution of tetraethylammonium perchlorate as exfoliation medium (Zu et al., 2019). The exfoliated phosphorene was used as the electrode in a new supercapacitor which exhibited excellent electrochemical performance. Compared the cathodic exfoliation based on the organic solvent mentioned earlier, a cathode exfoliation to prepare few-layer BP in an aqueous solution of hexadecyltrimethylammonium chloride (CTAC) has been reported by Lou et al. (Luo et al., 2019a). The influences of applied voltage, temperature, and concentration of CTAC on the efficiency of cathodic exfoliation have been systematically demonstrated. The few-layer BP with thickness of 5–8 nm can be obtained within 20 s, indicating the rapid and effective exfoliation of BP by the cathodic exfoliation in aqueous solution. Table 2 summarizes the key parameters, results, and applications of the BP sheets prepared via anodic and cathodic exfoliation.

**Table 2:** Summary of the anode and cathode electrochemical exfoliation few-layer BP

Anodic or cathodic exfoliation	Electrolyte	Solvent	Working voltage	Lateral dimensions/Thickness	Application	Ref.
Anodic exfoliation	0.5 M NA_2_SO_4_	H_2_O	7 V	5-10 μm/1-5 nm	Field emitters device	(Erande et al., 2015)
Anodic exfoliation	0.5 M NA_2_SO_4_	H_2_O	7 V	0.5–30 μm/1.4–10 nm	Field-effect transistor, sensor and photodetector	(Erande et al., 2016)
Anodic exfoliation	0.1 M [EMIM][BF_4_]	MeCN	8 V	/∼3 nm	Photothermal stability	(Tang et al., 2017)
Anodic exfoliation	0.1 M [BMIM][PF_6_]	MeCN	8 V	0.5-5 μm/∼2 nm	All-optical modulators	(Wang et al., 2018b)
Anodic exfoliation	[EMIM][BF_4_]	MeCN	8 V	5.0 ± 2.0 nm/2.0 ± 1.2 nm		(Tang et al., 2018)
Anodic exfoliation	0.5 M H_2_SO_4_	H_2_O	3 V			(Ambrosi et al., 2017)
Cathodic exfoliation	0.5 M TBAP	DMF	-5 V	/0.76–0.79 nm	Sodium-ion batteries	(Huang et al., 2017)
Cathodic exfoliation	0.1 M TBA·HSO_4_	PC	-8 V	20.6 μm/3.7 ± 1.3 nm	FET	(Yang et al., 2018)
Cathodic exfoliation	1 M TEAP	PC	10 V	>10 μm/3.4 nm	Supercapacitors	(Zu et al., 2019)
Cathodic exfoliation	0.1 M TBA PF_6_	PC	−30 V/12 h	/2-7 nm		(Xiao et al., 2018)
Cathodic exfoliation	0.01 M TAA	DMSO	-5 V/10 min	10 μm^2^/1.1–3.7 nm	Inkjet printing; photodetector	(Li et al., 2018)
Cathodic exfoliation	0.5 M CTAC	H_2_O	−30 V/20 s	/5-10 nm		(Luo et al., 2019a)

[EMIM][BF_4_], 1-ethyl-3-methylimidazolium tetrafluoroborate; [EMIM][BF_6_], 1-butyl-3-methylimidazolium hexafluorophosphate; MeCN, acetonitrile; TBAP, tetrabutylammonium hexafluo-rophosphate; DMF, N,N-dimethyl formamide; TBA·HSO_4_, tetra-n-butyl-ammonium bisulfate; PC, propylene carbonate; TEAP, tetraethylammonium perchlorate; TBA PF_6_, tetrabutylammonium hexafluorophosphate; TAA, tetraalkylammonium tetrafluoroborate; DMSO, dimethyl sulfoxide; CTAC, hexadecyltrimethylammonium chloride.

Apart from anodic exfoliation and cathodic exfoliation methods, bipolar electrochemical exfoliation method for BP has been reported by Mayorga-Martinez et al. (Mayorga-Martinez et al., 2016) and Baboukani et al. (Baboukani et al., 2019). In 2016, Mayorga-Martinez et al. have found that BP particles could be the raw material for the preparation of 2D BP in the bipolar exfoliation, which to a certain extent could realize low cost, compared with the bulk BP used as the electrode in the anodic and cathodic exfoliation (Mayorga-Martinez et al., 2016). Then, the bipolar electrochemical exfoliation has been used by Baboukani et al. to prepare the few-layer BP in deionized water electrolyte. The obtained few-layer BP with lateral dimensions up to a few hundreds of nanometers could be well deposited on the stainless-steel feeding electrode and exhibited remarkable capacitive energy storage performance. The method is low cost and environment friendly, providing insight for future research on the exfoliation and application of 2D BP (Baboukani et al., 2019).

### Direct growth of few-layer BP

Although the aforementioned top-down methods of mechanical cleavage, LPE, and ECE can successfully prepare few-layer BP, they are insufficient for the preparation of large-area, high-quality samples required for the preparation of logic circuits. Hence, the direct growth of few-layer BP is a vitally important strategy. Therefore, chemical vapor deposition (CVD), pulsed laser deposition (PLD), and gas-phase transformation will be specifically discussed as direct growth methods for few-layer BP.

**Figure 5:**
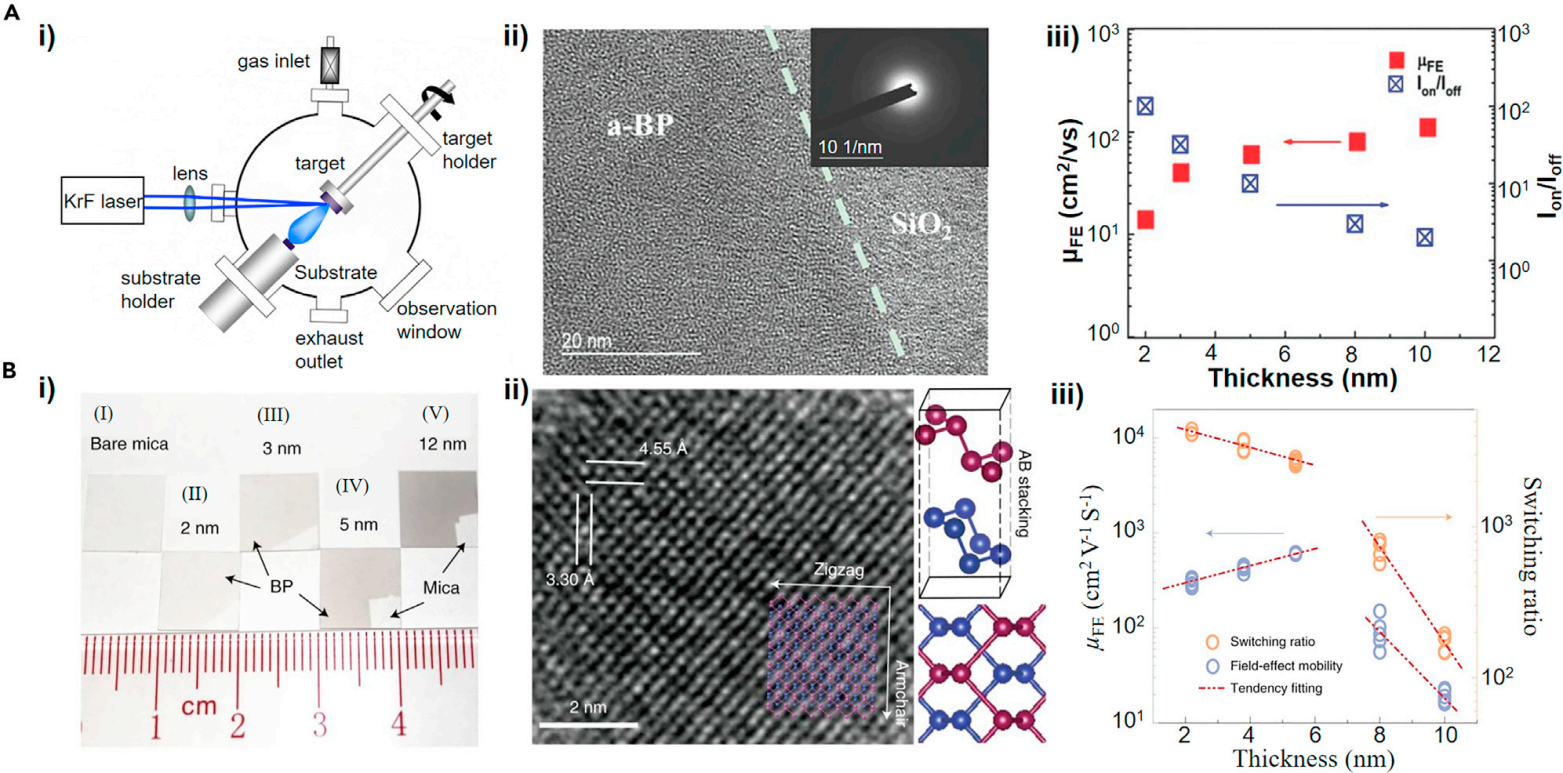
Direct growth of few-layer BP (A) (i) Schematic setup of PLD. (ii) High-resolution TEM image of an amorphous BP film with a selected area electron diffraction pattern (inset). (iii) Thickness-dependent field-effect mobility and the on/off ratio of the amorphous BP. (B) (i) Photograph of bare mica (I) and deposited BP films with different thickness from (II) to (V). (ii) High-resolution TEM image of the synthesized BP lattice, and the ball-and-stick schematics representing the adjacent two layers of BP from their different colors, whose lattice constants are 4.45 Å and 3.30 Å along the armchair direction and the zigzag direction, respectively. (iii) Thickness-dependent μ_FE_ and the switching ratio of the synthesized BP.

The direct synthesis of 2D BP via CVD was reported by Smith et al., in which amorphous red phosphorus was heated to 950°C and cooled at a temperature gradient of 50°C every 30 min in a tube furnace. The synthesized 2D BP possessed an average area >3 μm^2^ and a thickness of about 3.4 nm. Although the obtained BP is not ideal in size and insufficient for application in large-area electronic devices, this work is the first trial to prepare few-layer BP via CVD (Smith et al., 2016). In 2015, Yang et al. demonstrated a strategy using PLD (Figure 5A(i)) to produce wafer-scale amorphous BP ultrathin films. As depicted in Figure 5A(ii), the disordered structure of amorphous BP was illustrated via high-resolution transmission electron microscopy (TEM). The growth conditions of wafer-scale amorphous BP films have been systemically studied, by which the BP films could be deposited on Si/SiO_2_ substrate and graphene/copper. FETs based on the amorphous BP with a thickness of 2 nm possess a carrier mobility of 14 cm^2^ V^−1^ s^−1^ and moderate on/off current ratio of 10^2^, as shown in Figure 5A(iii) (Yang et al., 2015). Very recently, the synthesis of centimeter-scale and high-crystallinity BP film on mica substrate through improved conditions of PLD is reported by the same group (Figure 5B(i)). The experimental and theoretical simulation results show that the laser-activated plasma cloud creates extreme high-temperature and high-pressure conditions in the restricted area near the target surface, which is the key to the synthesis of few-layer BP film. In addition, the freshly exfoliated mica with atomic planarity and surface inertness as the substrate is also a factor in favor of the synthesis of BP film. Based on the factors mentioned earlier, BP with high crystallinity could be synthesized (Figure 5B(ii)). And Figure 5B(iii) depicts that the BP film with thickness of thinner than 5 nm on mica substrate was fabricated into top-gated FETs exhibiting mobility of 213 cm^2^ V^−1^ s^−1^ at room temperature (Wu et al., 2021). Hence, PLD is one method to produce large-area layered BP for logic circuits.

**Figure 6:**
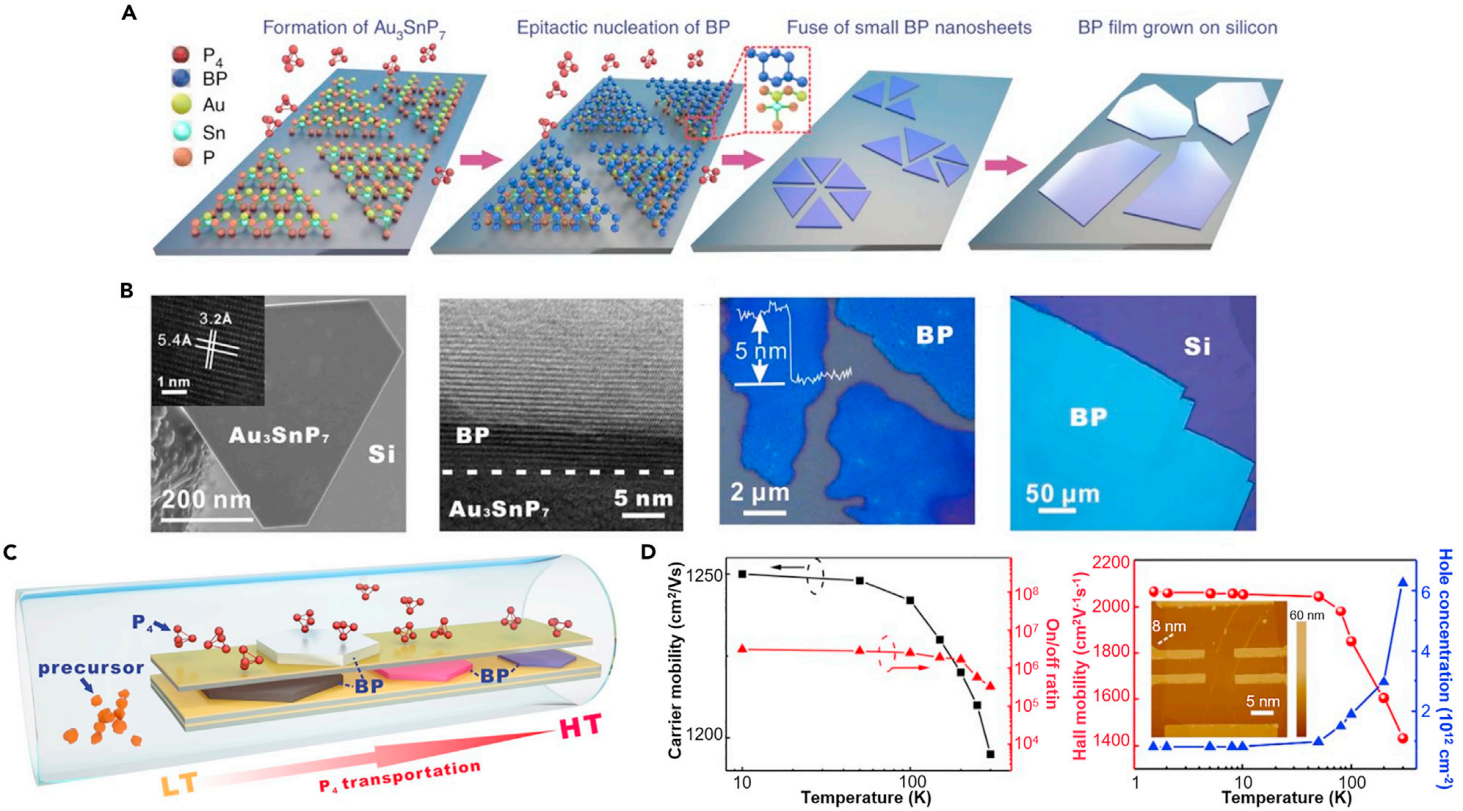
Gas-phase growth with epitaxial nucleation design of BP crystalline films (A) Schematic for the growth process of BP crystalline films including the formation of Au_3_SnP_7_, epitaxial nucleation of BP and fusing small BP nanosheets into large area films. (B) (Left one) SEM image of a Au_3_SnP_7_ nucleation seed grown on substrate with a high-resolution TEM image of the synthesized Au_3_SnP_7_ (inset). (Left two) Cross-sectional high-resolution TEM image of BP nanosheet grown on Au_3_SnP_7_. (Right two) Photograph of the transition state of fusing small BP nanosheets into large area films. (Right one) Photograph of the obtained BP film on substrate. (C) Schematic view of the experimental setup for the BP growth. (D) (Left) Temperature dependent carrier mobility and current on/off ratio of the obtained BP film. (Right) Temperature dependent μ_H_ (red line) and carrier concentration (blue line) of the BP film with an AFM image of its Hall-bar device (insert).

Recently, Xu et al. have proposed a novel growth strategy via gas-phase transformation to synthesize large-area, highly crystalline BP films with lateral dimensions up to several millimeters on silicon. Figure 6A illustrates the growth process, in which Au_3_SnP_7_ nucleation seeds are formed on silicon on which BP nanosheets subsequently grow, gradually fusing to form a final BP film. In the process, the Au_3_SnP_7_ is very important for the epitaxial nucleation and growth of BP film, whose thickness can be effectively controlled in the range of several nanometers to hundreds of nanometers (Xu et al., 2020). The characterization shown in Figure 6B demonstrates each stage of the synthesis of BP. As illustrated in Figure 6C, P_4_ molecules, originated from red phosphorus precursor, would transport from the low temperature (LT) side to high temperature (HT) side in a vacuum silica tube and finally crystalized into BP in between the stacked Si/SiO_2_ substrates coated with Au films. The amount of P_4_ molecules could influence the size and thickness of the BP, which could be effectively controlled through the transportation distance of P_4_ molecules and the space among the stacked substrates. The synthesized BP exhibited values of *μ*_FE_ and *μ*_H_ of over 1000 cm^2^ V^−1^ s^−1^ and 1400 cm^2^ V^−1^ s^−1^ at room temperature, respectively, and a high current on/off ratio of 10^6^, as shown in Figure 6D. Compared with the aforementioned top-down methods that cannot prepare large-area few-layer BP and PLD that cannot synthesize high-quality 2D BP crystals, gas-phase transformation can grow highly crystalline BP films with lateral dimensions up to several millimeters. The synthesized BP shows excellent electrical properties, suggesting great potential for applications in optoelectronic devices and logic circuits.

## Synthesis of BP quantum dots

Zero-dimensional BP quantum dots (BPQDs) also have ignited significant enthusiasm owing to their quantum confinement and edge effects. BPQDs have versatile advantages such as excellent photothermal conversion efficiency, a highly tunable bandgap, and large specific surface area, which make them promising candidates for various applications in areas including biomedicine, solar cells, and ion detection. The following top-down methods are currently used for synthesizing BPQDs: ultrasonic processing, solvothermal treatment, blender shearing, as well as laser irradiation.

**Figure 7:**
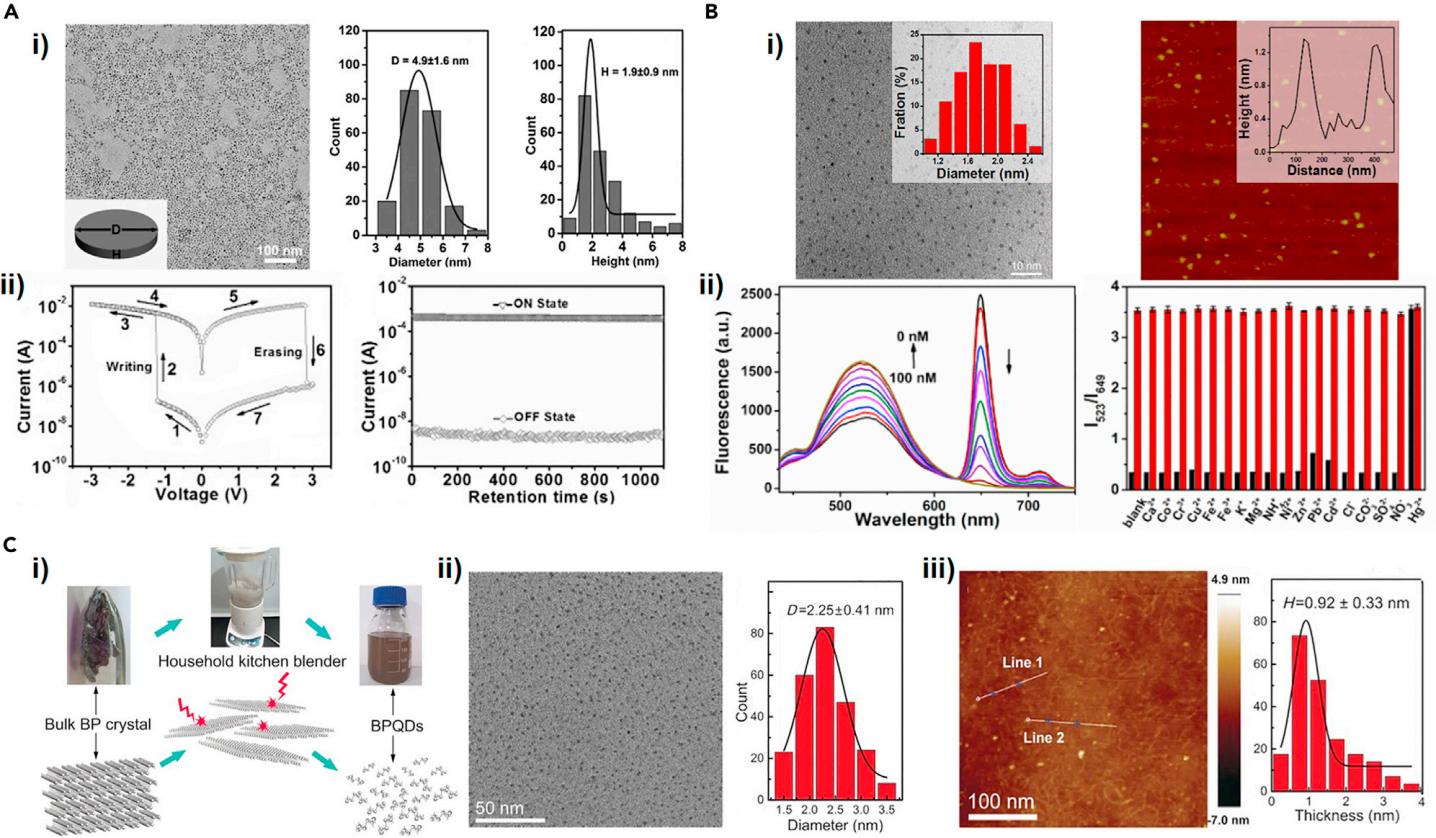
Synthesis and application of BPQDs (A) (i) TEM image of the synthesized BPQDs via a facile solution-based method (left). Statistical calculation of the dimension (middle) and the height profile (right) of the prepared BPQDs, in which D and H is average size and thickness. (ii) The output curve (left) and retention-ability measurement in the ON and OFF states (right) of a BPQD-based flexible memory device. (B) (i) (Left) TEM image of the as-synthesized BPQDs by the sonication-assisted solvothermal method and histogram of the dimension distribution of BPQDs (inset). (Right) AFM image of BPQDs with height profiles (inset). (ii) Fluorescence characteristic of the BPQDs-based assay for different concentrations of Hg^2+^ (left). Fluorescence characteristic of BPQDs-TPPS-Mn^2+^ system for different ions (right). (C) (i) Schematic for the process to obtain BPQDs via a household kitchen blender. (ii) TEM images of the as-prepared BPQDs (left) and histogram of the dimension distribution of BPQDs (right). (iii) AFM images of the BPQDs, in which white line1 and line2 referring to height profile line along their direction (left) and statistical calculation of the height profile of BPQDs (right).

In 2015, Zhang et al. first applied LPE to synthesize BPQDs with an average diameter of 4.9 nm and a mean thickness of 1.9 nm, as depicted in Figure 7A(i) (Zhang et al., 2015). The key condition for synthesizing BPQDs via LPE is to control the exfoliation time, compared with the preparation of nanosheets by the same method. In this work, the flexible memory device based on the prepared BPQDs in a polyvinylpyrrolidone (PVP) exhibited good flash memory effect and stability (Figure 7A(ii)). Sun et al. have successfully synthesized ultrasmall BPQDs with a mean diameter of 2.6 nm and an average thickness of 1.5 nm via a two-step process of probe and ice-bath sonication using NMP as solvent. The value of photothermal conversion efficiency for the obtained BPQDs can be up to 28.4%, indicating an excellent photothermal performance. Compared with liquid-phase ultrasonic exfoliation, a facile and controllable solvothermal treatment was also proposed to prepare ultra-small BPQDs on a large scale. By this method, the obtained BPQDs with an average diameter of 1.76 nm (Figure 7B(i)) were reported by Gu et al. who dispersed bulk BP in NMP and vigorously stirred the mixture for 12 h at 140°C under N_2_ atmosphere (Gu et al., 2017). The BPQDs were fabricated as a sensor for mercury ion detection with high selectivity and sensitivity, in which the sensor exhibited obvious fluorescence changes for different concentrations of Hg^2+^ (1–100 nM) and no changes in fluorescence response except for Hg^2+^, as shown in Figure 7B(ii). A method to prepare BPQDs by a household kitchen blender has been proposed by Zhu et al. (Zhu et al., 2016), as depicted in Figure 7C(i). The blender, as an easily accessible tool, can generate shear forces to exfoliate the bulk BP layer by layer to form BPQDs with a mean diameter of 2.25 nm and thicknesses of approximately 1 nm, as shown in Figure 7C(ii) and (iii). The BPQDs were fabricated as humidity sensors with excellent performance. Furthermore, the BPQDs also exhibit excellent photothermal conversion performance. This method cleverly uses the household kitchen blender to prepare BPQDs, which can also be applied for the quick preparation of quantum dots (QDs) from other layered materials. Ge et al. has prepared the phosphorene QDs (PQDs) with a mean size of about 7 nm by 20 min pulsed laser ablation of bulk BP in diethyl ether. The prepared PQDs exhibited good photoluminescence emission properties (Ge et al., 2016).

## Modification of BP

The material's instability is the other main disadvantage hindering the practical application of few-layer BP, besides of the lack of synthesis methods for large-area, high-quality samples. The preparation methods systematically introduced earlier yield few-layer BP samples of different sizes and properties, appropriate for applications in different fields. For example, the films with a lateral size of several micrometers and a thickness of several nanometers to tens of nanometers prepared by mechanical exfoliation are suitable for application in electronic and optoelectronic devices owing to inherently prominent electrical and optoelectronic properties. The few-layer BP obtained by LPE exhibits excellent performance for potential biomedical applications, such as controllable drug delivery and photothermal therapy (Qiu et al., 2019; Tang et al., 2020). The few-layer BP films synthesized via direct growth hold great promise for applications in large-area optoelectronic devices and logic circuits owing to their lateral dimensions of up to several millimeters. However, as mentioned, a further hindrance for practical applications of BP is its environmental instability. Related studies have shown that few-layer BP degradation divides into three steps: i) the emergence of superoxide anions on the surface under ambient light; ii) subsequent formation of dangling oxygen atoms making the surface highly hydrophilic; and iii) finally the breakdown of phosphorus bonds owing to interaction with the hydrogen bonds of water. Furthermore, the thinner the BP, the more accelerated its degradation (Zhou et al., 2016). Hence, it is vital to explore methods for preventing the degradation of BP, which include physical and chemical methods (Sang et al., 2019). Specifically, the physical method adopts the deposition of a relatively stable material on the BP surface to form a covering layer, preventing contact with ambient oxygen and water, such as to improve the stability of BP. Chemical methods can prevent BP from degradation by designing specific molecules or ions to recombine the active lone-pair electrons occupying the BP surface and isolating ambient oxygen and water. Here, such chemical methods will be introduced.

### Molecular modification

**Figure 8:**
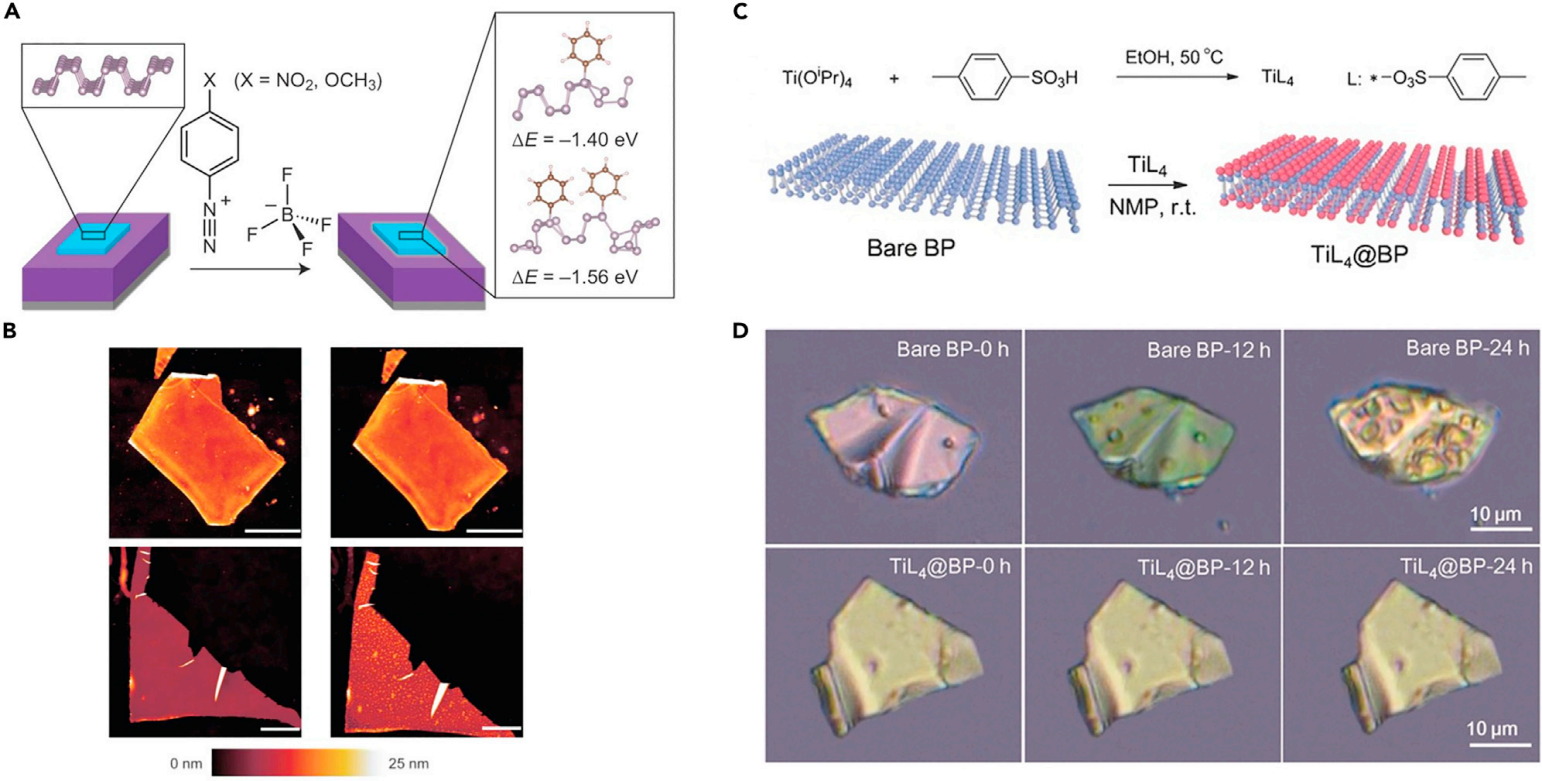
Molecular modification of BP nanostructure (A) Schematic for the reaction of benzene-diazonium tetrafluoroborate derivatives and the few-layer BP. (B) AFM image of a BP sheet immediately after functionalization with 10 mM 4-nitrobenzene-diazonium for 30 min (top-left) and the same sheet exposed for 10 days (top-right). An unmodified BP sheet (bottom-left) and the same sheet exposed for 10 days (bottom-right). Scale bars, 2 μm. (C) Reaction (top) and constitutional formula (bottom) of TiL_4_. (D) Optical images of bare BP (top) and TiL_4_ @ BP (bottom) sheets exposed for 0 h (left), 12 h (middle) and 24 h (right).

In 2016, Ryder et al. have proposed a covalent functionalization strategy to modify the few-layer BP using 4-nitrobenzene-diazonium and 4-methoxybenzenediazonium tetrafluoroborate salts, as illustrated in Figure 8A (Ryder et al., 2016). In this work, the morphology of the functionalized BP remained unchanged after 10 days of exposure to ambient conditions (Figure 8B), attributed to the phosphorus-aryl covalent bonds which effectively inactivated BP to react with ambient oxygen and water. FETs based on the modified BP exhibited better hole mobility and on/off current ratio than the unmodified BP. However, for the covalent functionalization strategy, it is difficult to control the progress of the reaction. Abellán et al. used 7,7,8,8-tetracyano-p-quinodi- methane and perylene bisimide to functionalize BP nanosheets (Abellán et al., 2016), which was effective in improving the stability of BP nanosheets. The results demonstrated a strong noncovalent interaction between the molecules and BP via theoretical calculations and experimental methods such as Raman spectroscopy, aberration corrected scanning TEM, and electron energy-loss spectroscopy. Zhao et al. investigated the stability of BP via coordination with titanium sulfonate ligands (TiL_4_) to form TiL_4_ @ BP, as demonstrated in Figure 8C (Zhao et al., 2016c). All X-ray photoelectron spectroscopy (XPS), Raman spectra, absorption spectra, photothermal performance measurements, and optical images demonstrated that TiL_4_ @ BP nanosheets exhibit good stability (Figure 8D) when dispersed in water and exposed to air for long time owing to the P-Ti coordination occupying the lone-pair electrons of phosphorus.

Although such surface passivation methods can achieve improved stability of BP, they also cause defects to some extent. A cleaner method for enhancing BP nanosheet stability was proposed by He et al. using vdWs epitaxial growth to deposit dioctylbenzothienobenzothiophene (C8-BTBT) thin films on BP (He et al., 2018). The Raman and roughness results of C8-BTBT-encapsulated BP were essentially unchanged for 20 days, manifesting that C8-BTBT thin films could effectively protect the BP. Specifically, FETs based on BP were fabricated using a nondisruptive electrode transfer method which ensured a highly clean contact interface and minimized interface trapping states (Liu et al., 2018a). After encapsulation with C8-BTBT, the FETs maintained high current for more than 8 days without significant degradation and exhibited an extremely high current density of 920 μA/μm and an on/off ratio of up to 1 × 10^7^ at room temperature. Besides, there is a research study based on density functional theory calculations and molecular dynamics simulations to study the passivation effect of perylenetetracarboxylic dianhydride (PTCDA) on BP demonstrating that 7-layer PTCDA can effectively protect BP and its edges (Zhao et al., 2017). Guo et al. systematically investigated the passivation effect of PTCDA on BP, demonstrating that PTCDA-covered BP can be sustained over 135 h without much degradation (Guo et al., 2019b). *In situ* UV photoelectron spectroscopy and XPS results confirmed the stability improvement. The method provides a strategy for improving BP stability by deposition of organic molecules with self-assembly capabilities.

### Ion modification

**Figure 9:**
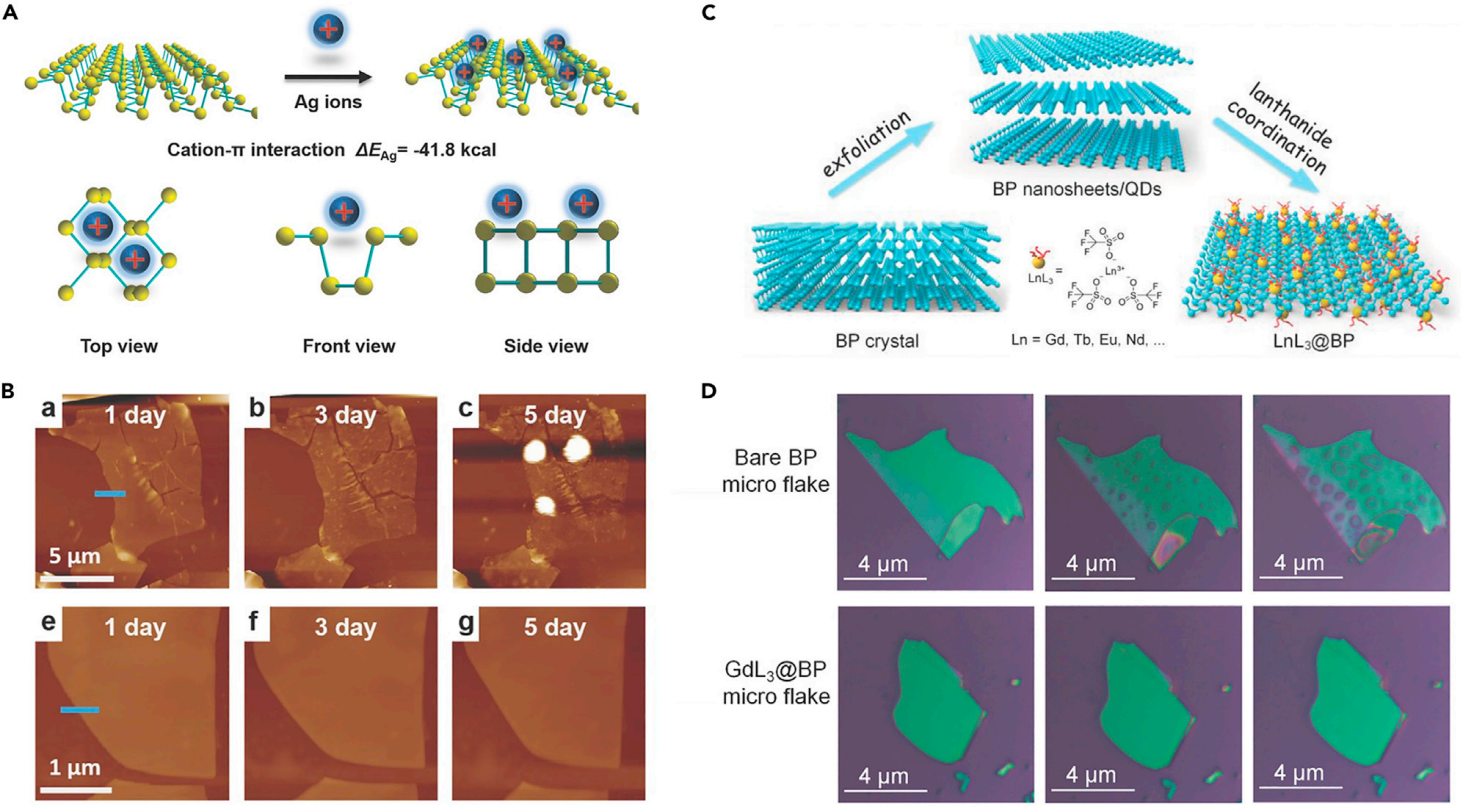
Ion modification of BP nanostructures (A) Schematic for Ag^+^ adsorbing on BP and their three different views. (B) AFM images of an unmodified BP sheet (top) and Ag^+^-modified BP sheet (bottom) exposed to air for 1 day, 3 days and 5 days. (C) Schematic diagram of the preparation of lanthanide-coordinated BP nanostructures and the formula of LnL_3_. (D) Photographs of a bare BP flake (top) and GdL_3_ @ BP flake (bottom) exposed to air for 0, 4 and 8 days.

The aforementioned molecular modification methods mainly work in a noncovalent form. In 2017, Guo et al. proposed a simple and effective metal-ion modification method using the cation-π interaction to improve the BP's intrinsic stability and its transistor performance (Guo et al., 2017). As shown in Figure 9A, metal ions would bind to the surface of BP with the conjugated π bond, which is the key factor to prevent the reaction between BP and the oxygen in the air. Silver-ion (Ag^+^)-modified BP can be well preserved from degradation for 5 days of exposure to air at 95% relative humidity (Figure 9B). The performance of a transistor based on Ag^+^-modified BP exhibited significant improvement in electrical properties, with a hole mobility of 1666 cm^2^ V^−1^ s^−1^ and an on/off ratio of 2.6 × 10^6^, which are 2 times and 44 times higher than that of bare BP, respectively. In addition to Ag^+^, the effect of Mg^2+^, Fe^3+^, and Hg^2+^ on the stability and transistor performance was also studied, demonstrating varying degrees of improvement due to different ions having different extranuclear electrons. In 2018, Wu et al. demonstrated a surface lanthanide-coordination strategy, which can effectively modify BP nanosheets or BPQDs, improving their stability and imparting new functions (Wu et al., 2018). In this work, lanthanide (Ln) ions (including Gd^3+^, Y^3+^, La^3+^, Nd^3+^, Sm^3+^, Eu^3+^, Tb^3+^, and Er^3+^) were prepared in the form of lanthanide trifluoromethesulfonate (LnL_3_). A surface Ln coordination process is shown in Figure 9C, in which the lone-pair electrons of phosphorus atom are coordinated with the empty orbital of the Ln ions. The color of the dispersion and the trend of the absorption spectra of GdL_3_ coordination BPQDs after 8 days of exposure have not changed significantly, demonstrating that GdL_3_ coordination can effectively improve the stability of BPQDs. As shown in Figure 9D, the morphology of a GdL_3_ coordination BP nanosheet was unchanged over 8 days, while large and dense droplets appeared on a bare BP sheet. Moreover, the lanthanide-modified BP possesses fluorescent properties and magnetic resonance imaging contrast owing to the existence of lanthanide ions and can be used as an efficient photothermal agent, giving the modified BP great application potential in biomedicine. The lanthanide trifluoromethesulfonate mentioned earlier is also a Lewis acid. Recently, Tofan et al. have systematically studied the effect of a series of commercial group 13 Lewis acids on BP (Tofan et al., 2021). Also, they have reported that AlBr_3_ Lewis acid could more effectively protect the few-layer BP from ambient degradation for at least 84 h and preserves transport properties of BP-based FETs for at least 72 h. Zhang et al. proposed a cationic cisplatin species, Pt-(NH_3_)_2_(NO_3_)_2_, coordinated with the BP nanosheets obtaining Pt @ BP, which has showed stability for 10-day exposure to ambient conditions (Zhang et al., 2019a). The Pt @ BP has exhibited potential biomedical applications owing to its good cellular uptake rate and significant increment in the aspect of the drug sensitivity of cisplatin-resistant cancer cell lines compared with unmodified BP. A comprehensive summary of related studies of molecular- and ion-modified BP with respect to their modification strategies and potential applications is presented in Table 3.

**Table 3:** A summary of the stability and potential applications of few-layer BP via molecular and ion modification

Material	Modification	Methods	Stability	Application	Ref.
BP sheets	Ag^+^-modified BP	Cation-π interactions	Morphology maintained for 5 days	FET	(Guo et al., 2017)
BP sheets or QDs	Lanthanide-coordinated BP	Ln-P coordination	Morphology maintained for 8 days	Photothermal performance; magnetic resonance imaging	(Wu et al., 2018)
BP flakes	Group 13 Lewis acids-functionalized BP	Lewis acidic binding to the surface of BP	Morphology maintained for at least 84 h.	FET	(Tofan et al., 2021)
BP nanosheets	Cisplatin-modified BP	Coordination between the bare BP and the platinum	Morphology maintained for 10 days	Photothermal performance	(Zhang et al., 2019a)
BP nanosheets	TCNQ- and PDI-functionalized BP	Noncovalent functionalization of BP			(Abellán et al., 2016)
BP flakes	Aryl-diazonium-functionalized BP	Covalent functionalization of BP	Morphology maintained for 10 days	FET	(Ryder et al., 2016)
BP nanosheets or QDs	Titanium-sulfonate-ligand-coordinated BP	P-Ti coordination	Morphology maintained for 24 h	Photothermal performance	(Zhao et al., 2016c)
BP flakes	Dioctylbenzothienobenzothiophene -encapsulated BP	van der Waals encapsulation	Morphology maintained over 20 days	FET	(He et al., 2018)
BP flakes	Perylenetetracarboxylic-dianhydride-molecules-encapsulated BP	Self-assembled molecules passivated BP	The modified device maintained for 135 h	FET	(Guo et al., 2019b)
BP nanosheets	Aromatic 1-pyrenylbutyric-acid-functionalized BP	Noncovalent π-π stacking interaction	Morphology maintained over 7 days	Cancer therapy	(Li et al., 2019c)
BPQDs	PFCz-NH_2_ covalently functionalized BPQDs	P-C bond between the polymer backbone and BPQDs	Absorption spectrum maintained for 60 days	Information storage	(Cao et al., 2019)
BP nanosheets	Hydroxyl-modified monolayer BP	Hydroxyl group occupying the lone pair of P		Photocatalytic carbon dioxide conversion	(Zhu et al., 2020)

BP, black phosphorus; BPQDs, BP quantum dots; FET, field-effect transistor; QDs, quantum dots; PFCz-NH_2_, poly[(9,9-dioctyl-9H-fluorene)-alt-(4-(9H-carbazol-9-yl)aniline)]; PDI, perylene diimide; TCNQ, 7,7,8,8-tetracyano-p-quinodimethane.

## Conclusions and perspectives

In this article, the basic structure and intrinsic properties of BP were first reviewed, highlighting the original reason for the attraction of widespread research interest in BP. As it is undoubtedly essential to obtain high-quality material for related research and applications, the synthesis methods of bulk BP crystal as an important raw material for preparing few-layer BP are second summarized. Subsequently, the research progress for popular top-down synthesis methods of few-layer BP is introduced, including mechanical exfoliation, LPE, and ECE. Direct growth strategies including CVD, PLD, and gas-phase transformation are also presented. In addition, the synthesis of important BPQD nanostructures and their unique properties have been systematically discussed. From the aspect of promoting the stability of few-layer BP, we finally recount various chemical modifications methods, including molecular modification and ion modification. Herein, various conclusions could be outlined from the progress on the preparation of few-layer BP. Briefly, according to the requirement of specific BP properties in different applications, the appropriate preparation methods can be selected.

In the potential application of biomedicine, which mainly focusses on controllable drug delivery and photothermal therapy for cancer treatment as listed in Table 4, LPE is currently the preferred method to prepare few-layer BP. This method can directly determine the lateral size and thickness of BP by controlling the different exfoliation parameters, such as exfoliation media, exfoliation time, and centrifugation time. For controllable drug delivery, few-layer BP is a promising candidate in which its own puckered honeycomb structure plays an important role. Moreover, few-layer BP obtained via LPE exhibits excellent photothermal effects because of its large near-IR extinction coefficient and high photothermal conversion efficiency.

**Table 4:** Characteristics of BP nanostructures in photothermal therapy and drug delivery

Fabrication methods	Lateral dimensions/thickness	η/*T*_max_	Drug loading capacity	BP component	Ref
Liquid-phase exfoliation	2.6 nm/1.5 nm	∼28.4%/31.5°C		BPQDs/PEG	(Sun et al., 2015)
Liquid-phase exfoliation	∼120 nm/1-2 nm	∼29.8%/∼50°C	DOX loading: ∼108%	BPNSs/PEGylated	(Tao et al., 2017)
Liquid-phase exfoliation		43.57%/65°C		BPNSs/Au	(Yang et al., 2017)
Liquid-phase exfoliation	∼200 nm/∼1 nm	36.0%/		BPNSs/PEI/siRNA	(Wang et al., 2018a)
Liquid-phase exfoliation	100∼200 nm/2.6 ± 1.5 nm	38.8%/∼45°C		BPNSs/hydrogel	(Qiu et al., 2018)
Ball-milling	3.2 ± 1.0 nm/1.2 ± 0.6 nm	36.8%/59°C		BPNPs/PEG	(Sun et al., 2016a)
Liquid-phase exfoliation	∼200 nm/∼5.5 nm	/45°C	DOX loading: 950%	BPNSs	(Chen et al., 2017b)
Liquid-phase exfoliation	200-300 nm/5.3 nm	/54.7°C	DOX loading: ∼8.2%	BPNSs@PDA-PEG-Apt	(Zeng et al., 2018)
Liquid-phase exfoliation	46.0 ± 3.2 nm/	/53.9°C	DcF@sPL loading: 93%	BPNSs-DcF@sPL	(Ou et al., 2018)
Liquid-phase exfoliation	∼20 nm/3-4 nm	/42.8°C	siRNA loading: 96%	BPNPs/PEG/PEI/siRNA	(Chen et al., 2018)

η: Photothermal conversion efficiency; *T*_max_: maximum light conversion temperature *in vitro* or *in vivo*.

PEG, polyethylene glycol; PEGylated, polyethylene glycol-amine; PEI, polyethylenimine; siRNA, small interfering RNA; DOX, doxorubicin; PDA, polydopamine; Apt, aptamers; BPNSs-DcF@sPL is chemo-phototherapeutic nanoplatform, in which D is doxorubicin, c is chitosan-polyethylene glycol, f is folic acid, s is small interfering RNA and PL is programmed death ligand.

To construct high-performance electronic devices, high-quality interfaces of few-layer BP are necessary, which would lead to a high carrier mobility and a promising switching performance. However, the electrical properties achieved vary according to different preparation methods. When mechanically exfoliated BP severed as the channel material, a high carrier mobility of 5200 cm^2^ V^−1^ s^−1^ (Long et al., 2016) and a high on/off ratio of 10^6^ (Si et al., 2017) were observed in BP-FETs at room temperature, exhibiting the intrinsic electrical properties of BP. The gas-phase transformation method is favorable to produce large-scale devices, in which the device channel would need to be several millimeters for logic circuit design. However, such devices showed a slightly lower carrier mobility of ∼1000 cm^2^ V^−1^ s^−1^ and an on/off ratio of ∼10^5^ at room temperature owing to the defect sites that formed during high-temperature growth.

In the field of optoelectronics, few-layer BP can be applied to photodetectors, modulators, and mode-locked lasers owing to its suitable bandgap and excellent nonlinear optical properties. Similar to electronic devices, few-layer BP obtained by mechanical exfoliation is also favored in photodetector applications. The bandgap of mechanically exfoliated few-layer BP varies with its thickness, which has a range from ∼5 nm to ∼200 nm, giving BP-based photodetectors a broadband photoresponse from near-IR (900 nm) to the terahertz band at room temperature. Furthermore, it was found that a modulator prepared by mechanically exfoliated BP can achieve a faster optical modulation speed of 113 ns. When applied to mode-locked lasers, few-layer BP, which can be prepared by LPE or mechanical exfoliation, exhibits a high repetition rate and a narrow pulse width in the range from near-IR (1064 nm) to mid-IR (2783 nm) owing to its excellent nonlinear optical properties. An integrated summary of studies of BP applied in electronics and optoelectronics is presented in Table 5.

**Table 5:** Properties of few-layer BP in electronics and optoelectronics

BP-based nanostructures	Fabrication methods	Lateral dimensions/thickness for BP	Application	Properties	Ref
BP nanosheets	Mechanical exfoliation	∼5 μm/5-10 nm	FET	On/off ratio: 10^5^ and hole mobility:∼1000 cm^2^ V^−1^ s^−1^ at room temperature	(Li et al., 2014)
BP nanosheets	Mechanical exfoliation	Several μm/∼5 nm	FET; CMOS inverter	On/off ratio: 10^4^ and hole mobility: 286 cm^2^ V^−1^ s^−1^ at room temperature; Gain of the CMOS inverter: 1.4	(Liu et al., 2014)
BP nanosheets	Mechanical Exfoliation	∼5 μm/∼9 nm	AlO_x_-encapsulated FET	On/off ratio: ∼10^3^ and hole mobility:∼100 cm^2^ V^−1^ s^−1^ for over 2 weeks in ambient conditions	(Wood et al., 2014)
BP nanosheets	Tip ultrasonication	Several μm/16-128 nm	FET	On/off ratio: ∼10^4^ and mobility:∼50 cm^2^ V^−1^ s^−1^ at room temperature	(Kang et al., 2015)
BP nanosheets	Liquid-phase exfoliation	<1 μm/	Flexible memory	On/off ratio:∼10^4^ and retention stability: over 10^5^	(Hao et al., 2016)
BP nanosheets	Mechanical exfoliation	/3–8 nm	Photodetector	Spectral range: visible-NIR; Responsivity: 4.8 mA/W and Response time: 1 ms under wavelength of 640 nm and 10 mW power	(Buscema et al., 2014)
BP nanosheets	Mechanical exfoliation	6.5 μm/11.5 nm	A waveguide-integrated photodetector	Responsivity: 135 mA/W and internal quantum efficiency of 10%; high response bandwidth exceeding 3 GHz	(Youngblood et al., 2015)
BP flakes	Mechanical exfoliation	∼10 μm/∼20 nm	Photodetector	Responsivity: 230 A/W; rise time and fall time: 4.8 ms and 6.8 ms at 1550 nm	(Liu et al., 2018b)
BP nanosheets	Mechanical exfoliation	/10-15 nm	Photodetector	Response wavelength range: 532 nm to 3.39 μm; Responsivity: 82 A/W at 3.39 μm	(Guo et al., 2016)
BP nanosheets	Mechanical exfoliation	/8 nm	Photodetector	Wavelength range: 400-900 nm; Responsivity: 7 × 10^6^ A/W at 20 K for a short channel 100 nm device	(Huang et al., 2016)
BP nanosheets	Mechanical exfoliation	/5 nm	hBN-sandwiched BP photodetector	Responsivity: 518, 30, and 2.2 mA/W; Noise equivalent power: 0.03, 35, and 672 pW Hz^−1/2^ at 3.4, 5, and 7.7 μm, respectively, at 77 K.	(Chen et al., 2017c)
BP flake	Mechanical exfoliation	/225 nm	hBN-encapsulated graphene-contact BP photodetector	Responsivity: 1.43 A/W, detectivity: ∼8.67 × 10^8^ cm Hz^1/2^ W^−1^ and NEP: ∼7 pW/Hz^−1/2^ at 3.4 μm; rise time and fall time: ∼1.8 ns and ∼1.68 ns at 1.55 μm	(Chang et al., 2020)
BP nanosheets	Electrochemical exfoliation	0.5–5 μm/∼2 nm	Modulator	Modulation depth of 17 dB and a rise time constant of 2.5 ms	(Wang et al., 2018b)
BP flake	Mechanical exfoliation	/40 nm	Waveguide-Integrated BP modulator	Modulation depth: ∼5 dB at -3′.85 μm; bandwidth: 400 kHz	(Li et al., 2019b)
BP flake	Mechanical exfoliation	/22 nm	All-optical modulator based on a phosphorene-assisted silicon micro-ring resonator	Rise time and decay time are only 475 and 113 ns, respectively; the 3 dB bandwidth of 2.5 MHz	(Cheng et al., 2020)
BP flakes	Mechanical exfoliation		Mode-locked laser	Modulation depth of 18.55%, and saturation intensity of 10.74 MW/cm^2^ at 1550 nm	(Chen et al., 2015b)
BP flakes	Mechanical exfoliation	/∼4.5 nm	Mode-locked laser	Modulation depth: 8% and saturable intensity: 0.35 MW cm^−2^; the pulse width of 7.54 ps and repetition rate of 13.5 MHz at 1085.58 nm	(Hisyam et al., 2017)
BP nanosheets	Liquid-phase exfoliation	439.3 nm/10.2 ± 1.5 nm	Mode-locked laser	Modulation depth: 18.5% and saturable intensity: 182 GW cm^−2^; the pulse duration of 635 fs and pulse repetition rate of 12.5 MHz centered at a wavelength of 1562 nm	(Xu et al., 2017)
BP flakes	Liquid-phase exfoliation	/∼16–25 nm	Mode-locked laser	Modulation depth: 0.67% and saturation fluence: 194 μJ cm^−2^; the pulse duration of 1.3 ps and pulse repetition rate of 290 MHz centered at 2094 nm	(Pawliszewska et al., 2017)
BPQDs	Liquid-phase exfoliation	2.1 ± 0.9 nm/	Mode-locked laser	Modulation depth: 36% and a saturable intensity: ∼3.3 GW cm^−2^ at 800 nm; a pulse duration of 1.67 ps at a wavelength of 1567.5 nm	(Xu et al., 2016)
BP nanosheets	Liquid-phase exfoliation	/0.6–2.0 nm	Mode-locked laser	Modulation depth of 10.9% at 1550 nm; pulse duration of 940 fs with central wavelength tunable from 1532 nm to 1570 nm.	(Luo et al., 2015)

BP, black phosphorus; BPQDs, BP quantum dots; NIR, near-infrared; FET, field-effect transistor; hBN, hexagonal boron nitride; NEP, noise equivalent power; CMOS, complementary metal oxide semiconductor.

In summary, BP has shown great potential in a wide range of applications owing to its unique structure and compelling properties. The preparation of large-area, high-quality, few-layer BP has been widely and deeply explored in recent years, and significant research results have been obtained. However, it is still a challenge to develop more facile synthesis methods of few-layer BP. As for the stability of BP, BP can be effectively preserved for several days without degradation via surface modification. However, the long-term stability of few-layer BP remains to be improved for practical applications. When the challenges of mass production and long-term stability are thoroughly overcome, few-layer BP will usher in the prospects of exciting new applications.
